# Supplementary material for: Bioconversion of Geniposide from Gardenia jasminoides via Levilactobacillus Enhancing Anti-Inflammatory Activity
Source: Foods. 2025 Dec 3;14(23):4156. doi: 10.3390/foods14234156 (PMC12692592; doi:10.3390/foods14234156)
Supplement: Supplementary file 1 [file foods-14-04156-s001.zip › foods-3984510-supplementary.pdf]

Supplementary Materials

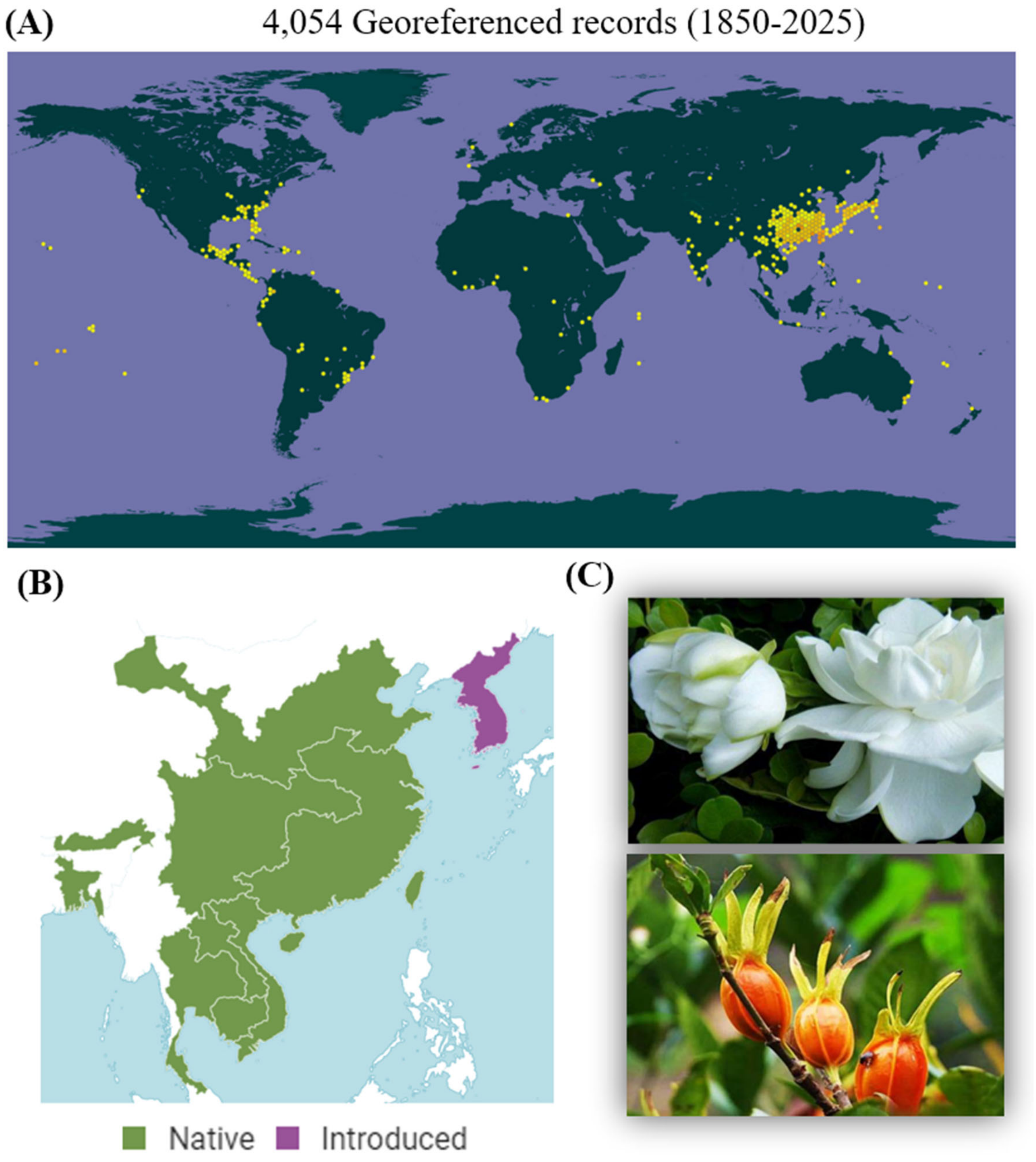

Figure S1. General introduction of *Gardenia jasminoides*. (A) Georeferenced records; (B) Origin of *Gardenia jasminoides*; and (C) Phenotype.

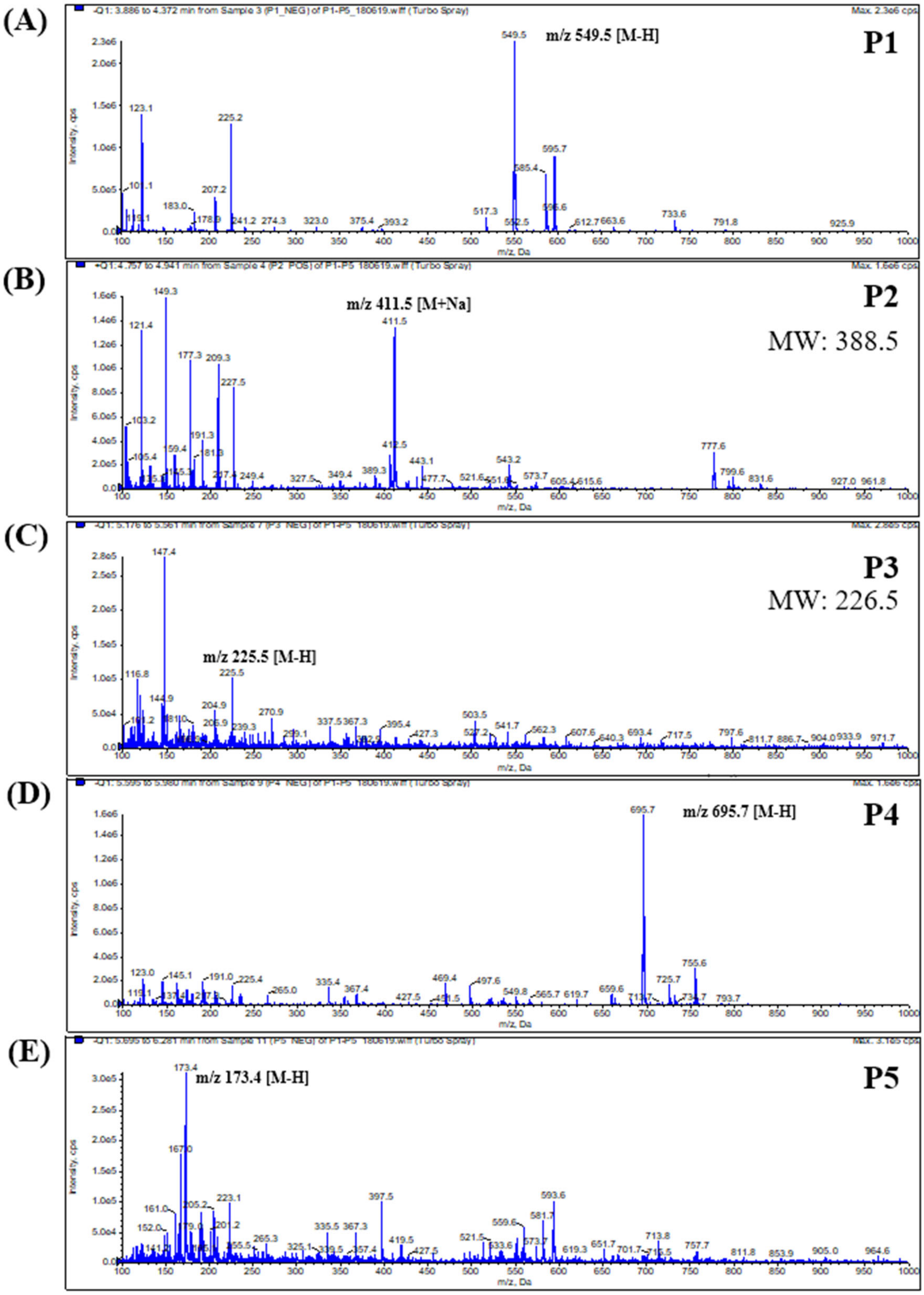

Figure S2. (A-E) LC/MS profiles of compounds P1–P5.

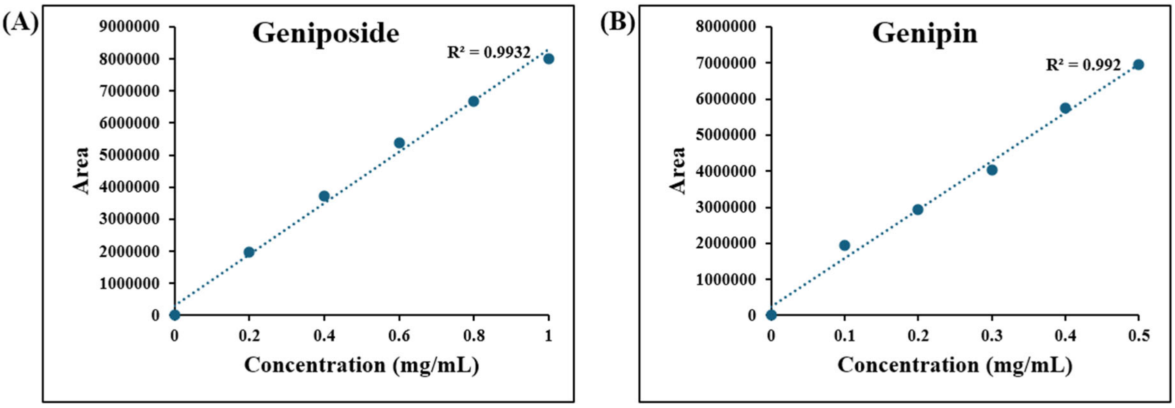

Figure S3. Standard curves of (A) geniposide and (B) genipin.

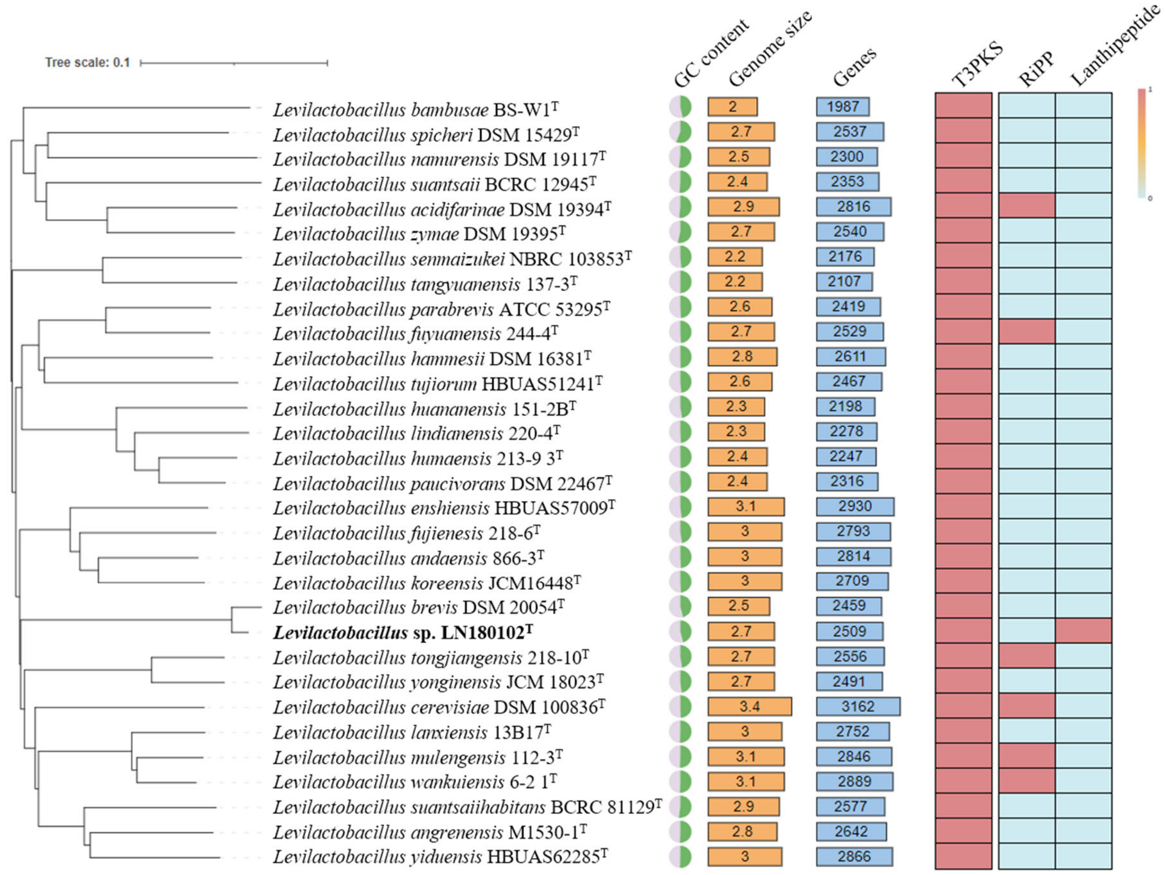

Figure S4. Phylogenomic analysis of *Levilactobacillus* sp. LN180102, genome information, and secondary metabolites prediction based on antiSMASH.

Table S1. List of selected 22 herbal medicinal plants.

No.	Herbal medicinal plants
1	*Artemisia campestris*
2	*Cassia obtusifolia* L.
3	*Castanea crenata*
4	*Chrysanthemum zawadskii* var.
5	*Cornus officinalis*
6	*Curcuma longa*
7	*Cuscuta japonica*
8	*Forsythia koreana*
9	*Gardenia jasminoides*
10	*Gastrodia elata*
11	*Illicium verum*
12	*Leonurus japonicas*
13	*Lonicera japonica*
14	*Lycium chinense*
15	*Momordica charantia* Linn
16	*Morus alba* L.
17	*Paeonia lactiflora*
18	*Patrinia villosa*
19	*Polygonatum sibiricum Redoute*
20	*Polygonum aviculare* L.
21	*Rehmannia glutinosa*
22	*Zizyphus zizyphus*

Table S2. Microbes or enzymes used for the bioconversion of *G. jasminoides* (1995 – 2025).

No.	Strain	Requirements	Conversion ratio	References
1	β-glucosidase	Enzyme immobilization<br>Enzyme incubation: 600 h	100%	Fujikawa *et al*, 1987.
2	*Eubacterium* sp. A-44		53%	Yang *et al*, 1995.
3	*Penicillium nigricans*	Microorganism culture: 120 h, 180 rpm, 30 °C<br>Medium volume 40%	95 %.	Xu *et al*, 2008.
4	β-glucosidase	Enzyme immobilization<br>Enzyme incubation: 2.5 h, 55 °C, pH 4.5	47.8%	Yang *et al*, 2011.
5	*Aspergillus niger*	Enzyme incubation: 24 h, pH 4.6, 45 °C, 20 U/mL, agitation	NM	Shon *et al*, 2012.
6	*Levilactobacillus casei* KFRI 127<br>*Levilactobacillus curvatus* KFRI 166<br>*Levilactobacillus confuses* KFRI 227	Microorganism culture: 48 h, 37 °C, pH 7.0 rearrange	16%	Lee *et al*, 2013.
7	*Trichoderma harzianum* CGMCC 2979	Microorganism culture: 30 °C, 150 r/min, 24 h<br>Enzyme incubation: 48 h, 30 °C, pH 6.1	97.8%	Dong *et al*, 2014.
8	*Aspergillus niger* Au0847	Enzyme incubation: 6 h, 30 °C	100%	Gong *et al*, 2014.
9	*Trichoderma reesei* QM9414	Microorganism culture: 34 h, 28 °C, 200 rpm<br>Enzyme immobilization	89%	Yang *et al*, 2018.
10	*Levilactobacillus antri*	Enzyme incubation: 4 h, 45 °C, pH 6.0	100%	Kim *et al*, 2017.
11	*Levilactobacillus plantarum* KFY02	$5*10^7$ CFU/kg (C57BL/6J mice food), 2mg/mL geniposide, 14 days	NM	Pan et al, 2020.
12	β-glucosidase	NM	NM	Xie et al., 2024.
